# Bio-Chemical Desensitization and Viscosity Reduction System for Ultra-Sensitive Heavy Oil Reservoirs in Jinjia Oilfield

**DOI:** 10.3390/molecules31142425

**Published:** 2026-07-10

**Authors:** Xiangyu Zhang, Ningkai Shu, Wangang Zheng, Hongguang Xu, Jing Hu, Zhongping Zhang, Shuaidong Wang

**Affiliations:** 1 College of Energy Resources, China University of Geosciences (Beijing), Beijing 102200, China; zhangxiangyu.slyt@sinopec.com; 2 Chunliang Oil Production Plant, Shengli Oilfield, Sinopec, Binzhou 256600, China; 3 Sinopec Exploration and Development Research Institute, Beijing 102200, China; snk.syky@sinopec.com; 4 Petroleum Engineering Technology Research Institute, Shengli Oilfield, Sinopec, Dongying 257000, China; xuhg90.slyt@sinopec.com (H.X.); hujing861.slyt@sinopec.com (J.H.); zhangzhp737.slyt@sinopec.com (Z.Z.); 5 Earth and Space Sciences Institute, Peking University, Beijing 102200, China; 2406395249@pku.edu.cn

**Keywords:** ultra-sensitive, heavy oil reservoir, biological-chemical desensitization and viscosity reduction, oil displacement mechanism, mobility control, synergistic application

## Abstract

The Jinjia oilfield in Shengli oilfield is a typical ultra-sensitive reservoir characterized by high crude oil viscosity, poor fluidity, high clay content, and weak cementation. During development, oil-sand mixtures readily plug pore throats. Various development methods including water flooding and thermal recovery have been implemented, yet severe problems persist: inability to inject, failure to displace, and lack of capacity to produce. To address these challenges, a functional microbial mineral-modified desensitization-chemical viscosity-reduction dual-effect agent, a self-growing gel dispersion profile control agent, and a low-damage deep acidizing system were developed. Laboratory experiments clarified the enhanced oil recovery mechanism of the bio-chemical desensitization and viscosity-reduction system. Results indicate that the desensitization and viscosity-reduction system can inhibit clay swelling, with the anti-swelling improvement rate of core permeability reaching 56%. Chemical viscosity reduction enabled heavy oil to “flow effectively,” achieving a viscosity reduction rate of 98.9% after adsorption. The profile control agent dispersed and migrated, then stably adsorbed onto particle surfaces to plug high-permeability channels, demonstrating strong anti-scouring capability and effectively suppressing channeling flow. In the composite system, bio-chemical desensitization and viscosity reduction synergistically enhanced mobility control, achieving an oil recovery factor of 56.5%, representing a 26.3% increase over post-water-flooding viscosity-reduction flooding. After two pilot well groups in the Jinjia oilfield were converted from water flooding to bio-chemical desensitization and viscosity-reduction composite flooding, single-well oil production capacity increased by 2.8-fold, water cut decreased by 12%, and both development performance and economic benefits were significantly improved—transforming the situation from “increasing water without increasing oil” to “increasing both liquid and oil production.” The research findings provide important reference value for the effective development of ultra-sensitive reservoirs.

## 1. Introduction

Globally, heavy oil resources are abundant, but sensitive heavy oil reservoirs—characterized by high clay content, strong water sensitivity, and high crude oil viscosity—have long been recognized as a worldwide challenge in the field of enhanced oil recovery. During the development of such reservoirs, clay swelling, particle migration, and asphaltene deposition are highly prone to occur, leading to a sharp decline in reservoir permeability. Conventional water flooding, thermal recovery, and chemical viscosity reduction methods often yield limited effectiveness due to poor adaptability. As a major producer of heavy oil, China faces this severe challenge as well. Taking the Shengli Oilfield as an example, its sensitive heavy oil reserves exceed 100 million tons. The reservoirs there have high clay content, strong sensitivity, and heterogeneity, along with high crude oil viscosity. During development, clay swelling and particle migration are observed. Various methods, including conventional development, cyclic steam stimulation, and viscosity reduction, have been successively applied, but the development results have been unsatisfactory.

To address the development challenges of sensitive heavy oil reservoirs, researchers worldwide have explored various technical approaches. According to their operating mechanisms, these methods can be broadly classified into four major categories: thermal recovery (e.g., cyclic steam stimulation, steam flooding, and in situ combustion), chemical recovery (e.g., viscosity reducer flooding, emulsification/viscosity reduction, and alkaline flooding), physical recovery (e.g., moderate sand production, ultrasonic viscosity reduction, and vibration-assisted recovery), and biological recovery (e.g., microbial viscosity reduction and microbial clay stabilization). Among them, thermal methods can effectively reduce crude oil viscosity but are constrained by reservoir thickness and heat loss; chemical methods lower interfacial tension and emulsify crude oil through surfactants or alkalis, yet their applicability is limited by clay mineral water sensitivity; physical methods such as moderate sand production can improve near-wellbore flow conditions, but they involve sand management risks; and biological methods show promise due to their environmental friendliness and in situ action potential. Focusing on these directions, many scholars have conducted specific studies on physical viscosity reduction, moderate sand production, emulsification/viscosity reduction, and related aspects. Lu Yanqiu et al. [1,2] analyzed the high-temperature alteration characteristics of strong water-sensitive heavy oil reservoirs in the Shengli oilfield. Cao Yanbin et al. [3,4,5,6,7,8] investigated enhanced oil recovery in extra-heavy oil or ultra-heavy oil reservoirs using steam-assisted methods. Liu Xiaoyu et al. [9,10] studied the impact of moderate sand production on low-permeability sensitive heavy oil reservoirs, considering it an effective development method to improve downhole seepage conditions. In recent years, replacing thermal viscosity reduction with biological and chemical viscosity reduction has become a research hotspot in chemical cold recovery of heavy oil. Emulsification viscosity reduction in heavy oil cold recovery is a key technological direction. Li Binfei et al. [11,12,13,14] studied the formation mechanisms, propagation, and enhanced oil recovery mechanisms of emulsions. However, the above-mentioned thermal, chemical, and physical methods all have significant limitations when applied to ultra-sensitive heavy oil reservoirs. Thermal methods are constrained by thin reservoir zones and heat loss, making it difficult to maintain effective thermal efficiency. Chemical methods are prone to causing secondary formation damage due to clay water sensitivity, and the effective duration of viscosity reducers is typically short. Physical methods, such as moderate sand production, may improve permeability in the short term, but they cannot fundamentally inhibit clay swelling and particle migration, and sand control remains challenging. Therefore, there is an urgent need to explore a novel technical approach that can address clay swelling at its source, while being both environmentally friendly and capable of providing long-term effectiveness. Petroleum microbiology technology exhibits unique potential and environmental advantages in inhibiting the hydration and swelling of clay minerals in reservoirs. Certain anaerobic iron-reducing bacteria can reduce Fe(III) in the montmorillonite structure to Fe(II), disrupting its lattice stability and promoting its transformation into non-expanding minerals, fundamentally inhibiting clay swelling [15,16]. Research by Cui et al. [17] found that four strains of iron-reducing bacteria achieved a 71% Fe(III) reduction rate in calcium-based montmorillonite under anaerobic conditions, significantly altering its mineral phase and reducing the montmorillonite interlayer spacing, resulting in a swelling reduction rate of 48.9%. This confirms an effective approach for preventing swelling through microbial mineral phase transformation. Mao Zhenqiang et al. [18] developed a low-cost, high-efficiency biological viscosity reducer suitable for the heavy oil in the Jinjia oilfield, achieving an enhanced oil recovery rate of 12–26%. Hu Xiaoli et al. [19] isolated thermophilic iron-reducing bacteria from high-temperature reservoirs in the Shengli oilfield. Under anaerobic conditions, these bacteria can also reduce structural iron in montmorillonite, promoting its transformation to illite, thereby reducing mineral swelling with a swelling reduction rate of 25.9%. This demonstrates the feasibility of applying indigenous thermophilic microorganisms in reservoirs for swelling prevention.

Taking the ultra-sensitive heavy oil reservoir in Shengli Jinjia oilfield as an example, this reservoir is characterized by high clay content, loosely consolidated reservoir rock, and high concentrations of resins and asphaltenes in the crude oil. Its water sensitivity index and velocity sensitivity index exceed 0.8 and 0.7, respectively. The composition of the oil sludge in storage tanks is complex, primarily consisting of minerals such as quartz and feldspar, clay minerals (16.8%), and asphaltenes. The interaction between asphaltenes in the porous media and solid particles detached due to velocity sensitivity increases the risk of reservoir plugging. For these reasons, the development of such reservoirs using a single water flooding approach can easily lead to ultra-sensitive permeability reduction. Furthermore, due to the thin reservoir thickness, a single thermal steam flood suffers from significant heat loss and low steam quality, resulting in poor thermal recovery performance. Currently, the average recovery factor is only 5.62%, with an oil production rate of 0.46%.

Aiming at the development challenges of ultra-sensitive heavy oil reservoirs, this study integrates biological desensitization and chemical viscosity reduction technologies to construct a bio-chemical composite system, and conducts research and application of water flooding conversion to bio-chemical composite flooding for sensitive heavy oil. The technical route is as follows: First, the sensitization mechanism of the reservoir is revealed through sensitivity evaluation and scanning electron microscopy analysis. Second, functional desensitization and viscosity reduction agents and self-growing gel dispersed profile control agents are developed, synergistically combined with screened sensitive mineral-modifying functional bacteria, to construct the bio-chemical composite system. Furthermore, through microscopic visualization and macroscopic core flooding experiments, the synergistic mechanism of the composite system in desensitization, viscosity reduction, and enhanced oil recovery is elucidated. Finally, key injection-production parameters of the system are optimized and field pilot tests are carried out. This research has established a bio-chemical desensitization, viscosity reduction, and profile control composite flooding technology system, providing theoretical support and practical reference for the efficient development of ultra-sensitive heavy oil reservoirs.

## 2. Results and Discussion

### 2.1. Water–Oil–Solid Coupled Sensitivity Mechanism in Ultra-Sensitive Reservoirs

To elucidate the water–oil–solid coupled sensitization mechanism of ultra-sensitive reservoirs, sensitivity experiments were conducted on reservoir samples to identify the sensitivity characteristics. Clay swelling experiments and laser particle size distribution analyses were performed to investigate the water–solid swelling effects. Zeta potential measurements and scanning electron microscopy (SEM) observations of inorganic particles before and after asphaltene adsorption were carried out to reveal the oil–solid aggregation mechanisms.

**Figure 1:**
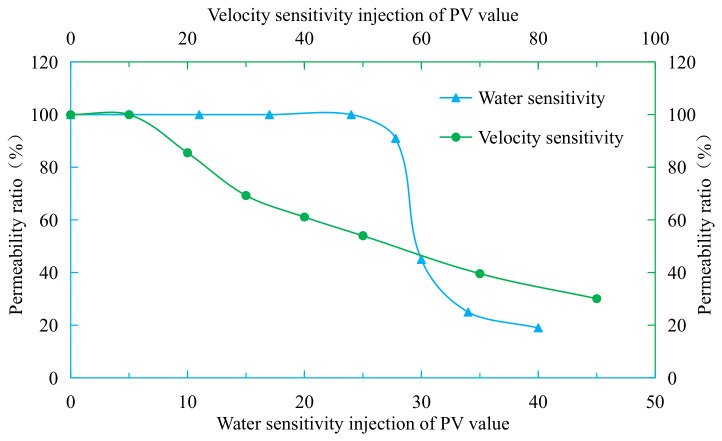
Water sensitivity and velocity sensitivity curves of reservoir in well jin 9-3-X3.

Figure 1 presents the water sensitivity and velocity sensitivity curves of the reservoir samples. As shown in Figure 1, the water sensitivity index of this reservoir was as high as 0.88, indicating strong water sensitivity. Meanwhile, due to the coupled effects of shallow burial depth, loose cementation, and the high viscous force of heavy oil, clay minerals were prone to detach and migrate with the fluid, causing particle plugging in porous media and resulting in significant velocity sensitivity with an index of 0.69. In summary, this block was a heavy oil reservoir characterized by both strong water sensitivity and strong velocity sensitivity.

**Figure 2:**
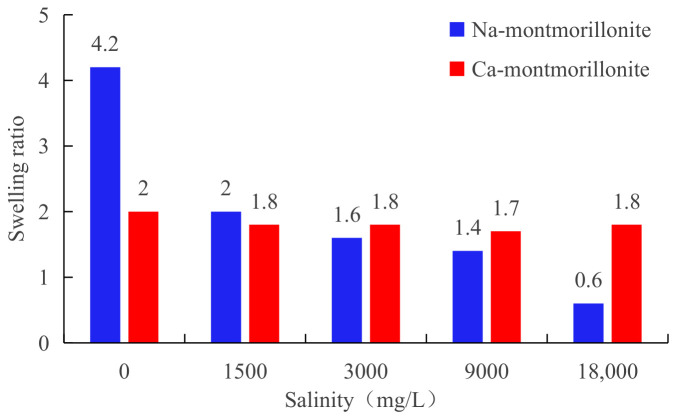
Bar chart of swelling coefficient vs. mineralization.

Figure 2 is a histogram illustrating the relationship between salinity and the swelling of calcium-based montmorillonite. Within the salinity range of 0 to 18,000 mg/L, the swelling multiple of calcium-based montmorillonite was relatively unaffected by salinity, remaining within the range of 1.7 to 2 times. The swelling multiple of sodium-based montmorillonite decreased with increasing salinity. When the salinity exceeded 9000 mg/L, the swelling multiple of sodium-based montmorillonite became lower than that of calcium-based montmorillonite. After water absorption and swelling, the montmorillonite structure became destabilized, providing a substantial number of migratory particles to the formation.

**Figure 3:**
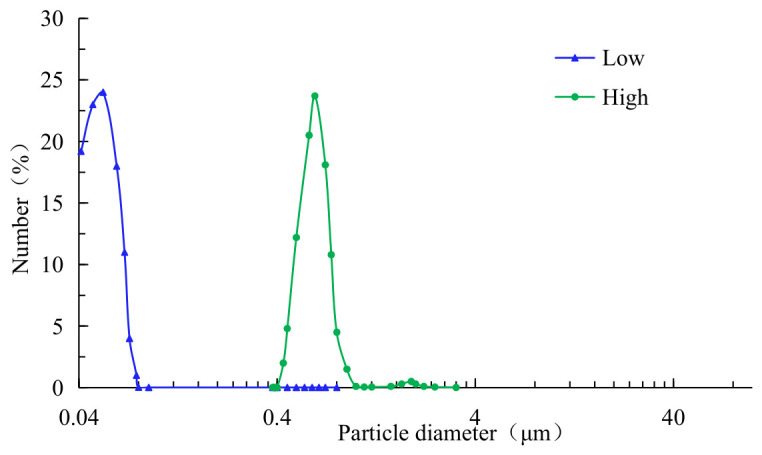
Laser particle size distribution spectrum of produced fluid.

Figure 3 presents the laser particle size distribution spectra of the produced water. It can be seen that the peak regions of migratory particle sizes in the post-water-flooding water samples were all below 2 μm, which were identified as montmorillonite fines. Such migratory particles could cause plugging at narrow pore throats.

**Figure 4:**
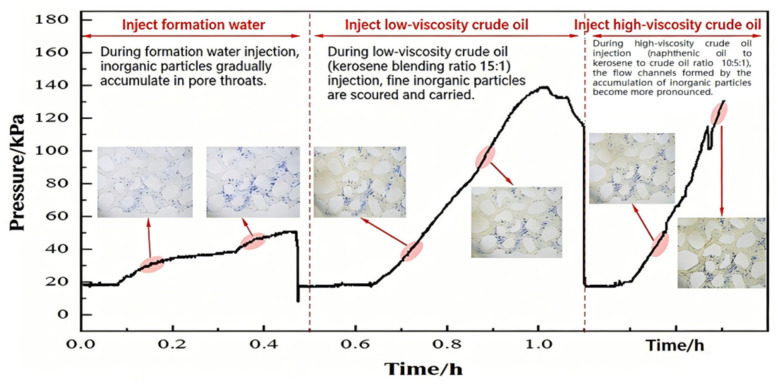
Impact of particles carried by fluids with different viscosities on pore throats.

Particle migration in the reservoir is influenced by factors such as injection rate and fluid viscosity. To clarify the water–oil–solid coupled sensitization mechanism in ultra-sensitive reservoirs, visual displacement experiments were conducted. Table 1 presents the data for particle threshold velocity under different fluid viscosities, and Figure 4 illustrates the impact of particles carried by fluids of varying viscosities on pore throats. It was observed that the higher the viscosity of the injected medium, the lower the threshold velocity for inorganic particles. Taking Ca-montmorillonite as an example, when the viscosity of the injection medium increased from 0.89 mPa·s to 560 mPa·s, the initiation flow rate decreased from 10 mL/min to 1 mL/min. This was attributed to the fact that the increased viscosity of the crude oil enhanced both the adhesive force and the drag force on the inorganic particles. Simultaneously, the high-viscosity fluid presented a greater obstacle to particle settling, making the particles easier to mobilize and migrate. On the other hand, the threshold velocity for quartz and feldspar was significantly higher than that for clay minerals, indicating that clay minerals were more readily mobilized and migrated.

During the injection of formation water, due to its relatively low viscosity, its carrying capacity was weak. A small number of inorganic particles accumulated at pore throats. Following this accumulation, the pressure gradually rose to 50 kPa, a relatively small increase. After the injection of low-viscosity crude oil (kerosene:crude oil ratio of 15:1), the carrying capacity increased due to the higher viscosity of the injected fluid. Fine inorganic particles within the pore channels were entrained and migrated by the crude oil, depositing and becoming retained in narrow pore throats. This led to an increase in injection pressure, reaching 140 kPa. The adsorption of crude oil onto the particles increased their effective size, blocking a wider range of pore throats and resulting in greater seepage resistance. When the crude oil viscosity was further increased (naphthenic oil:kerosene:crude oil ratio of 10:5:1), the flow paths formed by the accumulated inorganic particles became more pronounced. The frictional resistance during migration increased, causing the injection pressure to rise further to 150 kPa. Additionally, a phenomenon of sudden pressure drops was observed during the injection process. This occurred because, as the injection pressure increased, some of the blocked channels were forced open. However, as displacement continued, the oil–solid entrainment effect created new blockages in other pore channels, causing the pressure to rise again. This indicated a repetitive process of migration–plugging–remigration of particles in sensitive reservoirs.

**Figure 5:**
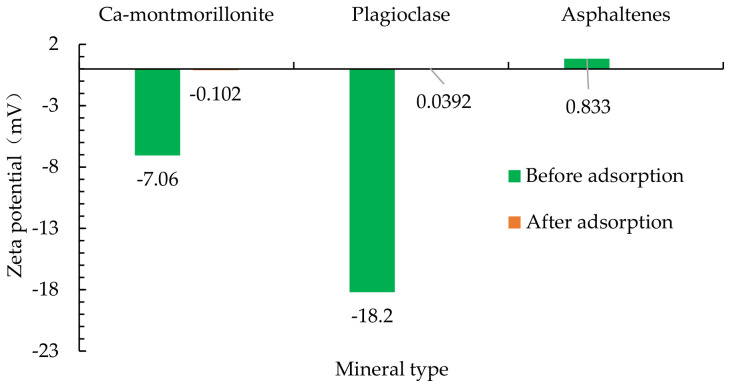
Bar chart of zeta potential before and after adsorption of inorganic particles and asphaltenes.

To further investigate the sensitivity mechanism of ultra-sensitive reservoirs, experiments involving zeta potential and particle size analysis were conducted. Figure 5 presents a histogram of the zeta potential of inorganic particles before and after asphaltene adsorption.

**Figure 6:**
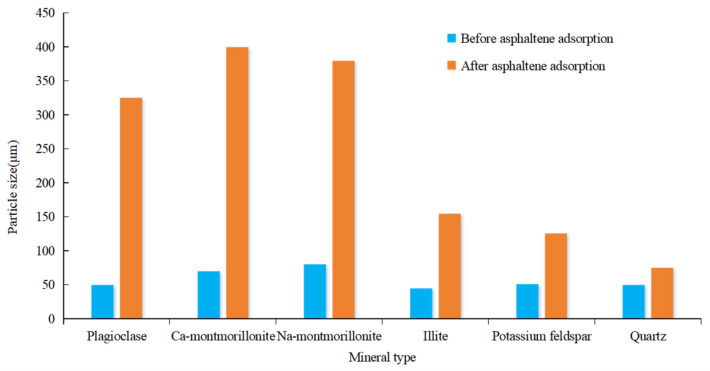
Bar chart of particle size changes after adsorption of asphalt on different mineral particles.

**Figure 7:**
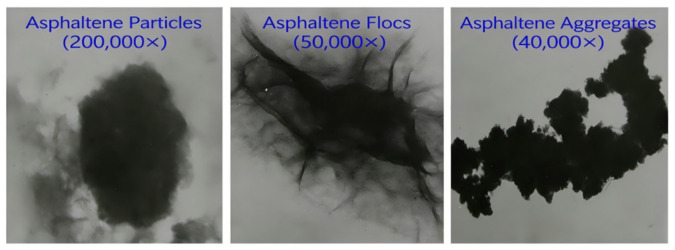
Flocculation and aggregation leading to enlargement of asphaltene particles.

Calcium-based montmorillonite carried a negative charge, whereas the asphaltene surface was positively charged. The zeta potential of calcium-based montmorillonite changed from −7.06 mV before adsorption to −0.102 mV after adsorption, indicating a decrease in electronegativity. This demonstrated that the differences in electrical properties and potential between mineral particles such as montmorillonite and asphaltenes led to their adsorption and flocculation. As shown in Figure 6 and Figure 7, after adsorbing asphaltenes, the particle size of different mineral particles increased by a factor of 1.4 to 6.2.

**Figure 8:**
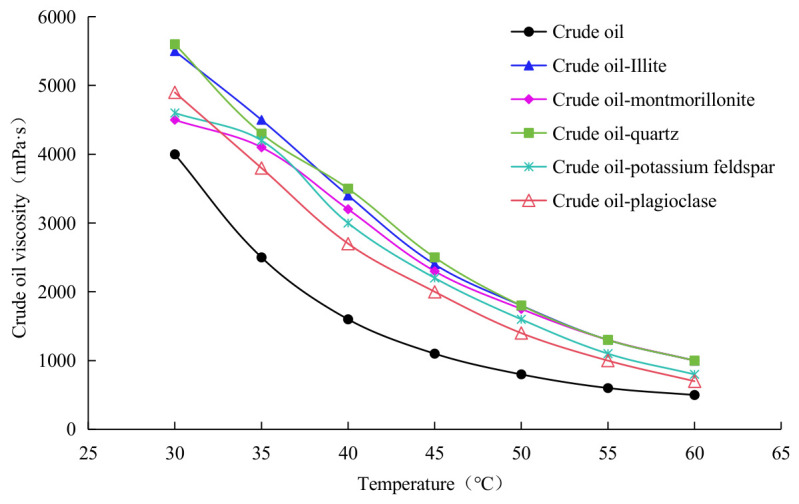
Viscosity–temperature curve of the crude oil–inorganic particle system.

Figure 8 shows that the viscosity of all systems decreases monotonically with increasing temperature. The crude oil viscosity drops from 4000 mPa·s at 30 °C to 400 mPa·s at 60 °C. Mineral adsorption elevates the viscosity at all temperatures, with the magnitude governed by both mineral type and temperature. The viscosity ratio (μ_oil-minerals_/μ_oil_) exhibits a pronounced non-monotonic profile, peaking in the 45–50 °C range. For the montmorillonite system, the ratio increases from 1.38 at 30 °C to a maximum of 3.20 at 45 °C, before declining to 2.25 at 60 °C. The illite, quartz, K-feldspar and albite systems reach maxima of 2.57 (50 °C), 2.20 (45 °C), 2.00 (45 °C) and 2.30 (45 °C), respectively. This phenomenon arises from the competition between adsorption layer structuring and thermal disruption. At low temperatures, the high bulk viscosity of crude oil masks interfacial effects. In the 40–50 °C window, the bulk viscosity drops sharply while adsorbed polar fractions (resins and asphaltenes) remain sufficiently bound to form a structured interfacial layer—mineral particles act as physical crosslinkers, causing a disproportionate viscosity increase. Above 50 °C, thermal desorption disrupts this network and the viscosity ratio decreases. This indicates that mineral adsorption most severely impedes oil mobility near 45–50 °C. The viscosity enhancement follows the hierarchy montmorillonite > illite > albite ≈ quartz > K-feldspar, which correlates directly with crystal chemistry. Layer silicates (montmorillonite and illite) possess 2:1 phyllosilicate structures with large specific surface areas, abundant surface Si-OH groups and high cation-exchange capacities, promoting strong adsorption of heteroatom-bearing asphaltenes and forming rigid interfacial shells; the expandable interlayer structure of montmorillonite further amplifies this effect. Framework silicates (quartz and feldspars) have smaller surface areas and fewer active sites, yielding weaker interfacial structuring and lower viscosity enhancement than layer silicates. Such oil–solid aggregation-induced viscosity enhancement can lead to pore throat plugging. In field applications, clay stabilizers, dispersants and viscosity reducers may be injected to weaken mineral–crude oil interfacial interactions, overcome adsorption resistance and improve oil mobility.

### 2.2. Development of Bio-Chemical Composite Systems

Based on the systematic understanding of the water–oil–solid coupled sensitization mechanism in ultra-sensitive reservoirs, functional chemical agents were developed and a biogeochemical hybrid mobility-improvement technology was established. First, a non-sensitive, high-conductivity, graded sand-screening artificial wellbore wall was constructed in the near-wellbore zone through a mixed-displacement process to achieve near-wellbore reservoir protection. Second, low-damage deep acidization was performed on the plugged throats to restore reservoir seepage capacity. Subsequently, a one-agent dual-effect oil production and displacement technology was employed, in which functional bacteria modified mineral desensitization and chemical viscosity reduction acted synergistically to simultaneously eliminate sensitivity and reduce crude oil viscosity. Finally, self-growing gel dispersion profile control agents were injected into the prone-to-channeling injection-production strips to achieve auxiliary uniform permeability and mobility control. Through this integrated approach, an innovative biogeochemical composite mobility-improvement technology was formed.

#### 2.2.1. Bio-Chemical Desensitization and Viscosity-Reducing Agent

**Figure 9:**
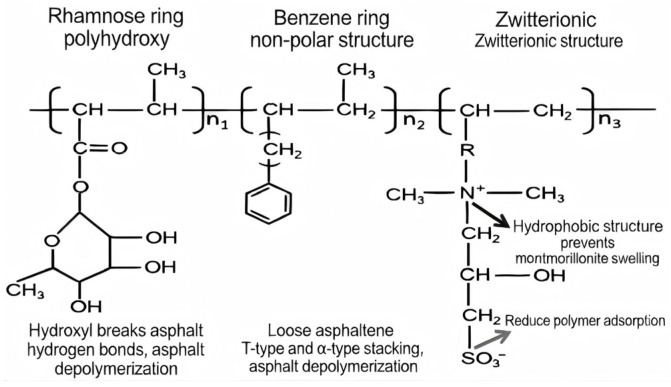
Molecular structure of bio-chemical desensitizing and viscosity-reducing agent.

In response to the mechanisms of montmorillonite structural instability due to swelling, asphaltene-particle adsorption and flocculation, and viscosity increase-induced sensitivity in sensitive reservoirs, a three-segment polymer structure was designed. This structure incorporated functional groups for biological bacterial desensitization, chemical depolymerization, and anti-adsorption. Figure 9 shows the molecular structural formula of the bio-chemical desensitization and viscosity-reducing agent. The alkane main chain and benzene ring side chains were lipophilic, while the rhamnolipid and small-carbon-number alkyl betaine groups were hydrophilic. By regulating the ratio of these functional groups, the HLB value was controlled between 12 and 16. The agent adsorbed at the oil–water interface, forming a stable, small-droplet oil-in-water emulsion. The hydroxyl groups disrupted asphaltene hydrogen bonds, and the non-polar structure of the benzene ring loosened the T-shaped and Π-shaped stacking of asphaltenes. The combined action of these groups achieved asphaltene depolymerization. The cationic groups in the amphoteric monomers inhibited montmorillonite swelling, while the sulfonic acid groups inhibited polymer adsorption.

**Figure 10:**
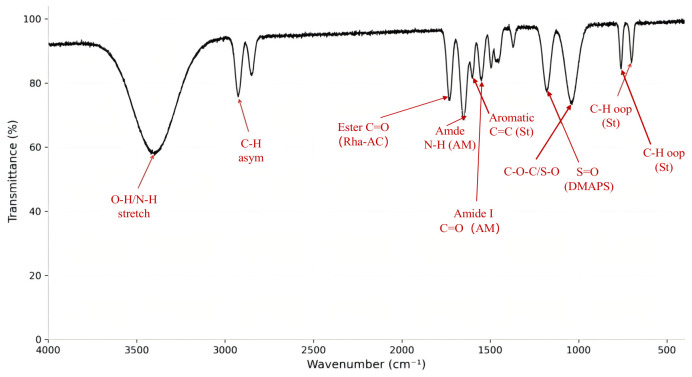
Infrared spectrum of P (AM-Rha-St-DMAPS).

The quaternary free radical solution copolymerization was carried out by one-shot feeding method, with rhamnosyl acrylate (Rha-AC), styrene (St), 2-(methacryloyloxy)ethyl dimethyl-(3-sulfopropyl)ammonium betaine (DMAPS), and acrylamide (AM) copolymerized at designed ratios (75%, 12%, 8%, and 5%, respectively). The polymerization procedure followed the bio-chemical desensitization and viscosity-reduction agent synthesis steps described in Section 3.2. The structure of the system was characterized by FT-IR spectroscopy, as shown in Figure 10. The broad peak at 3400 cm^−1^ corresponds to the overlapped stretching vibrations of multiple hydroxyl groups on the rhamnose ring and amide N-H of AM, confirming the successful incorporation of the sugar moiety. The double peaks at 1730 cm^−1^ and 1650 cm^−1^ are assigned to ester carbonyl (Rha-AC) and amide carbonyl (AM), respectively, demonstrating the coexistence of two carbonyl-containing monomers. The bands at 1600, 1495, 760, and 700 cm^−1^ in the benzene ring fingerprint region confirm the successful polymerization of styrene units. The characteristic peaks at 1180 and 1040 cm^−1^ for sulfonic groups verify the introduction of the DMAPS zwitterionic structure. This spectrum completely validates the successful integration of three functional structures in the trifunctional integrated polymer: rhamnose polyhydroxyl groups, styrene hydrophobic benzene rings, and DMAPS anti-swelling zwitterionic moieties.

Under conditions of wide oil–water ratios (8:2 to 2:8), broad viscosity-reduction concentration windows (0.2–0.5%), and high montmorillonite content (9.8%), six novel reservoir desensitization heavy oil viscosity-reduction flooding agents with different polymerization degrees were evaluated. Using the anti-sensitivity rate, interfacial tension, and post-adsorption viscosity reduction rate as primary indicators, Agent No. 5 was selected as the optimal desensitization and viscosity-reduction agent. Its anti-swelling improvement rate of permeability reached 56%, adsorption rate was 1.19%, interfacial tension was 1 × 10^−3^ mN/m, natural settling dehydration rate was 94.4%, viscosity reduction rate was 99.1%, post-adsorption viscosity reduction rate was 98.9%, and oil washing rate was 68%, as shown in Table 2.

**Figure 11:**
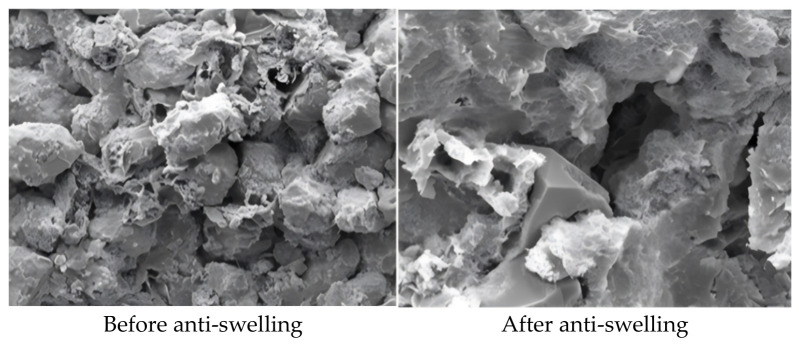
Scanning electron microscope images before and after anti-swelling treatment.

Figure 11 presents scanning electron microscope (SEM) images before and after anti-swelling treatment. A large quantity of clay minerals was observed adhering to the surfaces of sandstone particles, exhibiting a velvety and honeycomb-like distribution. This specific distribution state of illite–smectite mixed layers caused the particles to swell and exfoliate integrally with increasing water content or upon invasion by foreign fluids, thereby blocking pore throats and resulting in a sharp permeability decline. After the anti-swelling treatment, the resistance to fluid erosion was enhanced. A stable protective film formed on the surface of the clay particles, which preserved or even improved the reservoir’s physical properties.

#### 2.2.2. Self-Growing Gel Dispersion Profile Control and Displacement Agent

**Figure 12:**
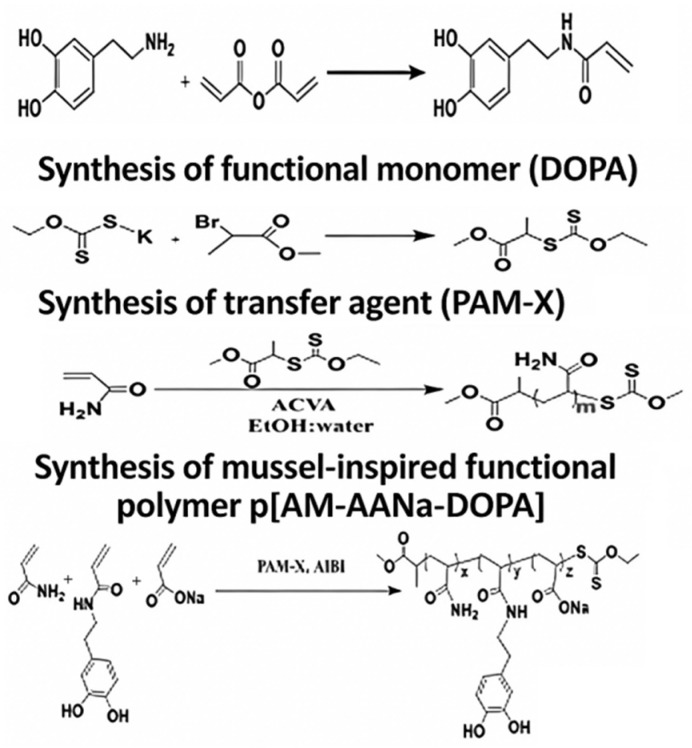
Synthesis of self-growing mussel-inspired functional polymer.

To address the issues of loosely consolidated reservoirs and high permeability in localized zones that easily lead to water channeling, a self-growing gel system was developed. A mussel-inspired polymer, CFPAM (Figure 12), exhibiting good adhesion, cohesion, and self-growing properties, was synthesized through the polymerization of acrylamide, sodium acrylate, and catechol-structured monomers. The catechol-structured monomer imparts underwater adhesion and protein-like self-healing functionality. Subsequently, this mussel-inspired functional polymer, CFPAM, was crosslinked with phenolic resin to produce spherical self-growing gel dispersion particles ranging from 0.3 μm to 60 μm. The synthesis procedure of the system followed the self-growing gel dispersion profile control agent synthesis steps described in Section 3.2.

**Figure 13:**
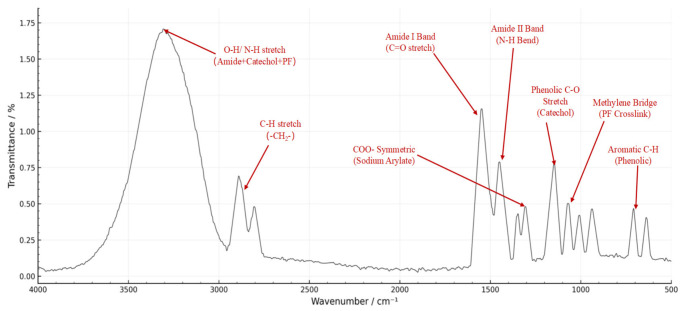
Infrared spectrum of self-generating gel dispersion drive agent.

The structure of the system was characterized by FT-IR spectroscopy, as shown in Figure 13. The FT-IR spectrum of CFPAM-PF microspheres exhibited characteristic absorption peaks of all structural components, confirming the successful crosslinking between the mussel-inspired functional polymer and phenol-formaldehyde resin. The broad peak near 3400 cm^−1^ was assigned to the overlapped stretching vibrations of amide N-H, catechol O-H, and phenolic resin O-H, indicating the formation of an extensive hydrogen bonding network within the system. The amide I and II bands at 1650 cm^−1^ and 1550 cm^−1^ verified the polyacrylamide backbone structure, while the symmetric stretching peak of carboxylate at 1410 cm^−1^ corresponded to the sodium acrylate component. The phenolic C-O stretching vibration at 1260 cm^−1^ demonstrated the successful incorporation of catechol moieties, and the methylene bridge vibration peak at 1180 cm^−1^ served as the signature evidence for the formation of phenol-formaldehyde crosslinking structures. Furthermore, the aromatic C-H out-of-plane bending modes at 830 cm^−1^ and 760 cm^−1^ further confirmed the existence of substituted benzene ring structures from both catechol and phenolic resin units.

These gel particles coalesced within large pore channels, forming a three-dimensional structure with interwoven large and small pores. The viscoelastic modulus of the gel decreased slowly with increasing aging time, indicating stable viscoelasticity. Influenced by zeta potential, the self-growing gel dispersion dispersed under low salinity conditions and coalesced under high salinity conditions. Under reservoir conditions, during 90 days of aging, the spherical particles gradually coalesced to form a bulk structure, enhancing shear resistance and demonstrating sustained, good mechanical strength.

#### 2.2.3. Supporting Low-Damage Acidizing Plug-Removal System

**Figure 14:**
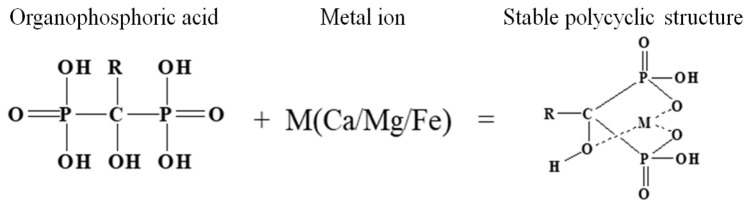
Long-acting acidizing system with chelated metal ions.

In response to formation damage caused by drilling mud filter cake blockage and the accumulation of silt and fine sand, a low-damage deep acidizing system was developed. This system utilizes low-sensitivity acid dissolution and modification to resolve blockages caused by drilling mud filter cake and clay minerals. The system incorporated 2% organic phosphonic acid chelating agent, which chelated metal ions, reduced the concentration of free Fe^3+^, formed a multi-ring stable structure (Figure 14), and achieved stepwise ionization, thereby retarding the acid–rock reaction rate. Additionally, 3% anti-sludging agent was added to prevent the disruption of the dispersed heavy oil morphology during acidizing, which could otherwise form sludge. This resulted in a low-damage, deep-acting effect that combines reservoir protection with retarded deep penetration. Simultaneously, it enhanced the synergistic effect of multi-stage filter cake building, establishing a non-sensitive, high-conductivity, graded sand-retention artificial borehole wall.

**Figure 15:**
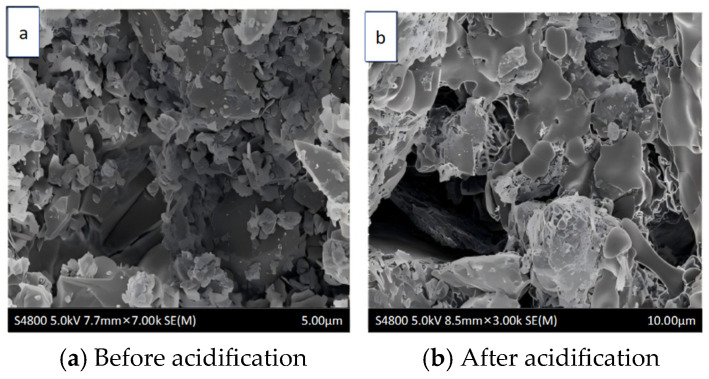
Scanning electron microscope images before and after acidification.

The low-damage acidizing plugging removal system was employed to treat the core. SEM images (Figure 15) after acidization revealed significant pore enlargement and channel unblocking effects, while simultaneously inhibiting secondary precipitation. Permeability experiments before and after acidization demonstrated that the core permeability increased from 196 mD prior to acidization to 598 mD after displacement with 10-fold pore volume acid solution, achieving a 3.05-fold permeability enhancement. This realizes deep dissolution and modification of ultra-sensitive heavy oil reservoirs.

#### 2.2.4. Breeding of Mineral-Modifying Functional Bacteria and Mechanism of Water-Sensitivity Mitigation

**Figure 16:**
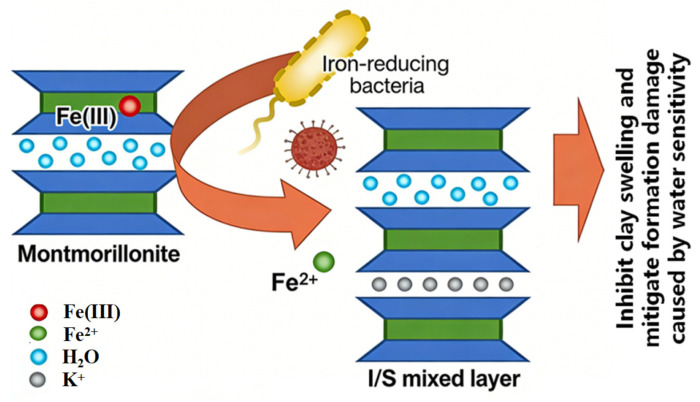
Desensitization mechanism of mineral-modified bacteria on sensitive minerals.

The pore throat blocking effect caused by the interlayer swelling of montmorillonite and its mixed-layer minerals upon water contact significantly reduced reservoir permeability, which was a bottleneck constraining enhanced oil recovery. To address reservoir damage caused by clay mineral swelling, particularly montmorillonite, a mineral-modifying and water-sensitivity-mitigating bacterial strain was selected. This strain was derived from the formation water of the target reservoir and anaerobically acclimated under simulated reservoir temperature conditions. After acclimation, the functional strain could promote the phase transformation of montmorillonite to illite by reducing Fe^3+^ in the montmorillonite crystal structure, thereby fundamentally resolving the water-sensitive swelling problem caused by high clay content in the reservoir (Figure 16).

**Figure 17:**
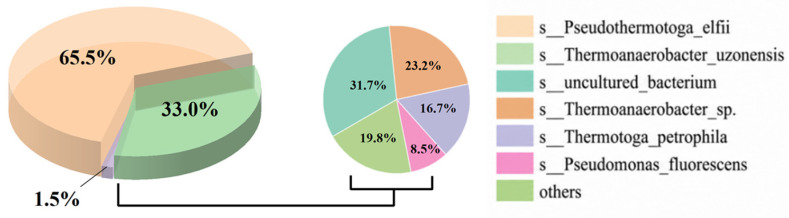
IRB-Mnt microbial species composition.

The isolated mineral-modifying functional bacteria were subjected to paired-end sequencing of the V3–V4 region of the 16S rRNA gene. After the raw sequences were generated, a series of quality-control steps were performed, including paired-end read merging, low-quality read filtering, and chimera removal, to obtain high-quality effective sequences. Subsequently, OTU-based clustering was carried out for species annotation and community structure analysis. The results (Figure 17) showed that the dominant taxa in the mineral-modifying functional bacterial community were pseudothermotoga elfii (65.5%) and thermoanaerobacter uzonensis (33%). These strains remained active under the high-temperature (65 °C) and slightly acidic (pH 6.5) conditions and were capable of significant interfacial reactions and biogeochemical interactions with montmorillonite.

**Figure 18:**
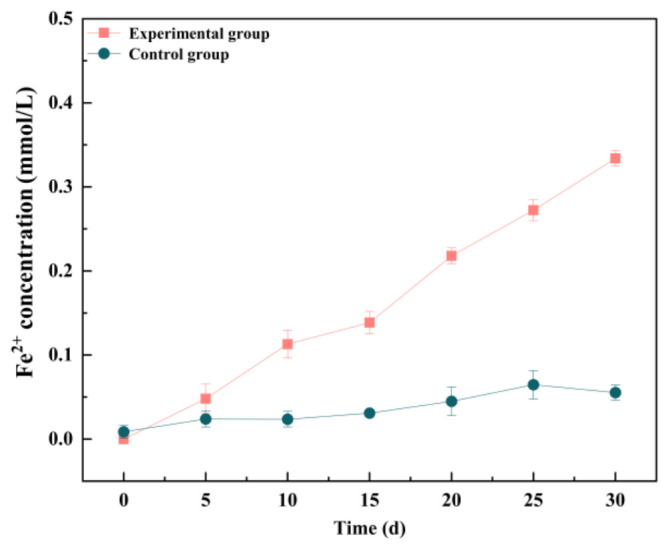
Changes in Fe^2+^ concentration and changes during functional microbiota interaction with montmorillonite.

Under simulated reservoir conditions at 63 ± 2 °C, with disodium nucleotide as the sole phosphorus source in the culture medium, the iron-reducing mixed culture IRB-Mnt was incubated with core samples for 30 days. The Fe^2+^ concentration in the liquid phase increased significantly from an initial value of 0 mmol/L, reaching 0.334 mmol/L in the IRB-Mnt experimental group (Figure 18), corresponding to a reduction efficiency of 37.49% for structural Fe(III) in montmorillonite. The increase in Fe^2+^ concentration indicates that the iron-reducing bacteria reduced structural Fe(III) in montmorillonite through their metabolic activities and released it into the solution system as Fe^2+^ ions. These results demonstrate that microbial metabolic activities promoted the acid-assisted reductive dissolution of structural Fe(III) in montmorillonite via acidification.

**Figure 19:**
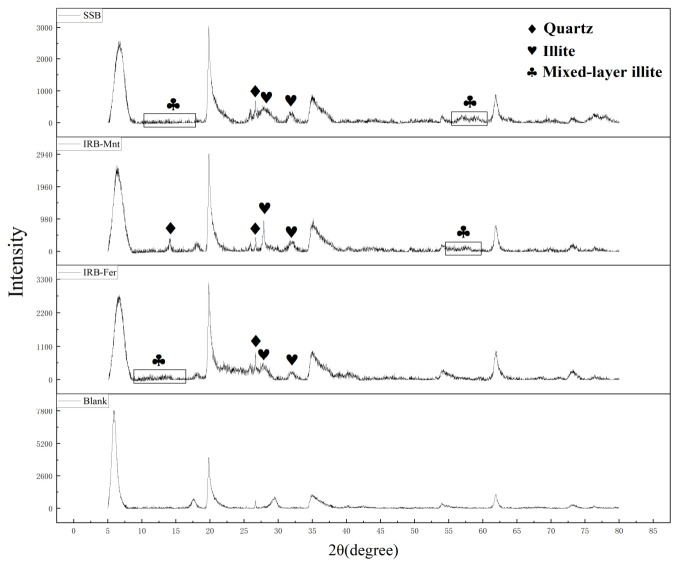
XRD patterns of reservoir core samples after interaction with functional microbiota: full-range XRD pattern.

After the modification treatment, the XRD patterns (Figure 19) showed that the characteristic peak width of the treated samples near 2θ = 25° increased, and the single mineral phase peak underwent splitting, indicating a decrease in the interlayer ordering of montmorillonite and possible disturbances in the interlayer structure as well as the local crystal lattice. Meanwhile, some of the peak positions corresponded to the characteristic diffraction peaks of illite or illite–smectite mixed-layer minerals, suggesting that an illitization trend or enhanced illite–smectite mixed-layer structural features may have emerged in the system.

After treatment with the modifying bacterial consortium, the swelling rate of montmorillonite decreased significantly. For core samples with montmorillonite contents as high as 21–35%, the anti-expansion improvement rate of core permeability after water sensitivity treatment reached 62%, and the cores with higher montmorillonite contents exhibited better swelling-reduction performance after treatment. These results indicate that the functional modifying consortium can effectively reduce the swelling propensity of clay minerals, thereby substantially mitigating reservoir damage caused by water sensitivity.

### 2.3. Oil Displacement Mechanism of Bio-Chemical Desensitization and Viscosity-Reducing Agents

Based on the newly developed bio-chemical desensitization and viscosity-reducing agent and the self-growing gel dispersion system, physical simulation experiments on enhanced oil recovery by desensitization and viscosity-reducing composite flooding were conducted. The oil displacement mechanism of the novel desensitization and viscosity-reducing agent was elucidated.

#### 2.3.1. Oil Displacement Mechanism of Bio-Chemical Desensitization and Viscosity Reducing Agent

**Figure 20:**
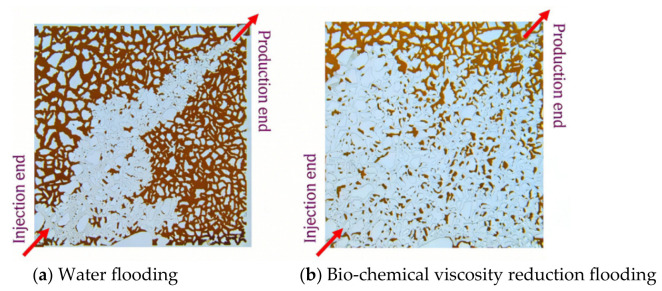
Distribution of remaining oil after water flooding and bio-chemical viscosity-reducing flooding.

Figure 20 shows the distribution of remaining oil after water flooding and bio-chemical desensitization and viscosity-reducing flooding. The viscosity-reducing flood rapidly emulsified and dispersed the large oil blocks remaining after water flooding, forming fine oil-in-water small oil droplets. The flow resistance of these small oil droplets was significantly reduced, allowing them to easily pass through the core pores and throats and be swept out by the displacing fluid. This substantially enhanced the oil washing efficiency in the swept area. The pore throat sizes remained unchanged, indicating a favorable desensitization effect.

**Figure 21:**
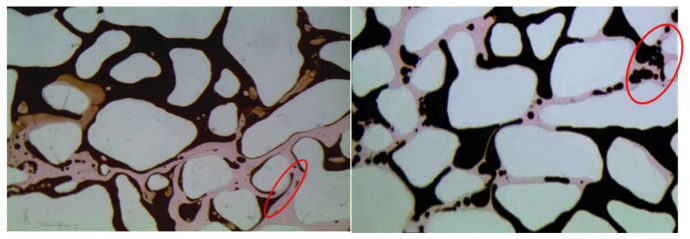
Deformation and filamentary stripping of remaining oil (**left**) vs. aggregation and stacking of oil-in-water emulsion (**right**).

From the left image of Figure 21 the deformation, elongation, and stripping of remaining oil during bio-chemical desensitization and viscosity-reduction flooding can be observed. During the viscosity-reducer flooding, the system significantly reduced the oil–water interfacial tension, substantially softening the oil–water interfacial film. This enabled the previously immobile, isolated heavy oil, after multiple dynamic contacts with the viscosity reducer, to be readily elongated and deformed under flowing shear forces, gradually stretching into oil threads and subsequently breaking into very fine droplets. This effectively mobilized the entire isolated residual oil, reduced residual oil saturation, and thereby enhanced heavy oil recovery.

From the right image of Figure 21 the accumulation and packing of oil-in-water emulsions during the same flooding process can be observed. The emulsified droplets formed by the viscosity reducer accumulated and packed together as they flowed through pore throats, gradually occupying the flow channels in the porous media. With the continuous generation, migration, stacking, and accumulation of emulsion droplets, the flow paths in the pore throats narrowed, and flow resistance increased. The clusters formed by the accumulated droplets increased the migration resistance of the droplets themselves. To a certain extent, this also increased the injection resistance for the subsequent displacing fluid, causing the follow-up fluid to be diverted into unswept areas and thereby improving the sweep efficiency.

#### 2.3.2. Displacement Mechanism of Self-Growing Gel Dispersion System

**Figure 22:**
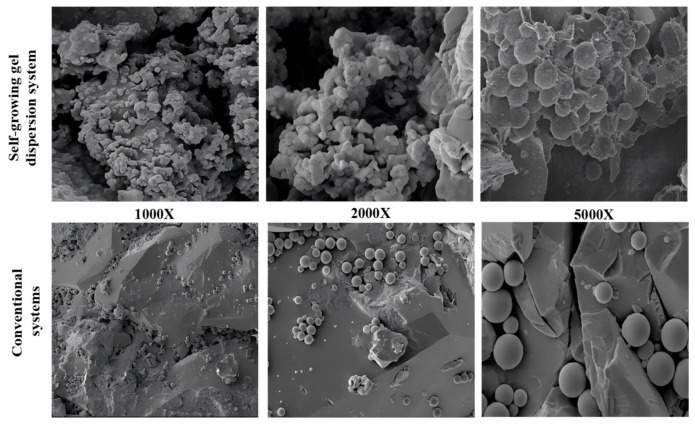
Distribution pattern of the system on rock surface.

**Figure 23:**
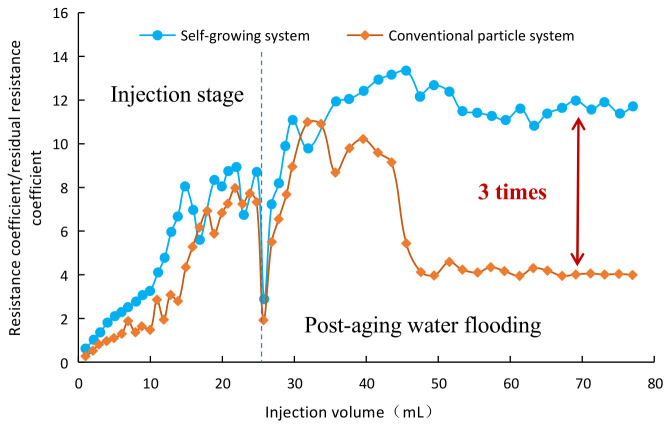
Resistance coefficient/residual resistance coefficient curve.

Compared with conventional systems, the self-growing gel dispersion, after bio-chemical desensitization and viscosity reduction, underwent long-distance dispersion and migration, increased in cohesion, and stably adsorbed onto particle surfaces, effectively blocking large pore channels. Its performance was significantly superior to that of conventional systems (Figure 22). During the injection phase, the resistance coefficient of this system was higher than that of conventional particle systems. In the water flooding stage after aging, the residual resistance coefficient of the self-generating gel dispersion system reached 12, while that of the conventional particle system was approximately 4. It was three times that of the conventional system and indicated strong anti-erosion ability, enabling effective long-term maintenance of the sealing effect (Figure 23).

**Figure 24:**
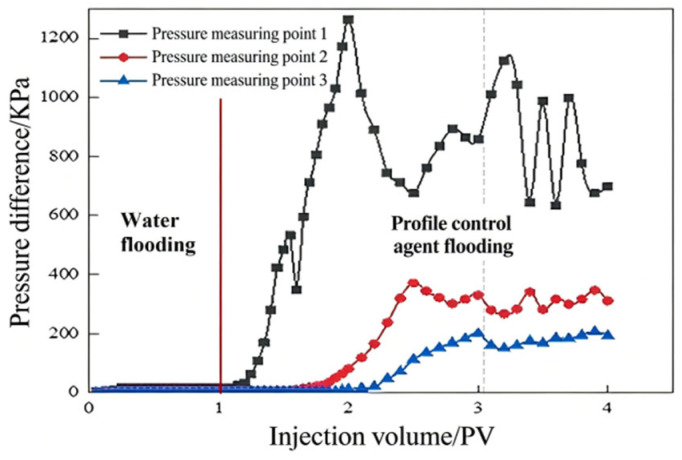
Pressure variation curve at different measurement points in long core.

Figure 24 shows the pressure curve of the novel bio-chemical desensitization and viscosity-reducing composite flooding experiment. According to the test results, when the system concentration exceeded 1.0%, the overall plugging rate was greater than 80%. As the injected pore volume increased, the pressure at the distal end of the core gradually rose and stabilized, reaching a stable seepage state where profile control was achieved without causing blockage.

During water flooding, the recovery factor was 19.8%. After water flooding, the viscosity-reducing flooding achieved a recovery factor of 30.2%. After water flooding, the novel viscosity-reducing composite flooding system achieved a recovery factor of 56.5%. The new composite flooding system exhibits synergistic effects of anti-swelling, viscosity reduction, and profile control, resulting in a higher recovery factor—an increase of 36.7% over water flooding and 26.3% over post-water-flooding viscosity-reducing flooding.

**Figure 25:**
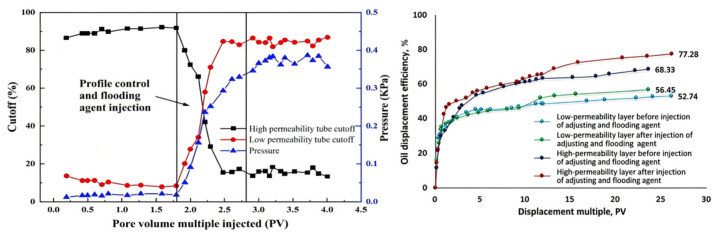
Flow fraction and oil displacement efficiency curve in dual-tube long core oil displacement experiment.

Figure 25 presents the fractional flow and oil displacement efficiency curves of the dual-tube long core flooding experiment. When the core permeability ratio was close to 5, after 1.75 PV of water flooding, the fractional flow in the high-permeability tube rapidly decreased to 10%. At this point, the novel bio-chemical desensitization and viscosity-reducing composite flooding system was injected. Upon injection, the pressure initially rose and then stabilized. The fractional flow in the low-permeability tube gradually increased to 80%. This system exhibited the characteristics of plugging high-permeability zones while displacing oil from low-permeability zones, achieving an improvement rate in the water injection profile of over 80%. After the profile control and displacement process, the oil displacement efficiency in the high-permeability and low-permeability tubes increased by 3.71% and 8.95%, respectively, resulting in an overall increase of 12.66%.

#### 2.3.3. Mineral Modification Patterns of Bio-Chemical Desensitization and Viscosity-Reducing Agents

**Figure 26:**
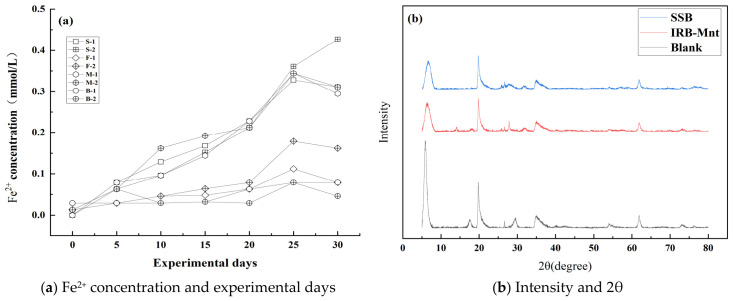
Fe^2+^ concentration and XRD characteristics after treatment with mineral-modified bacteria.

Under simulated reservoir temperature conditions, an expanded reaction system involving functional bacterial strains and mineral powder was established. Figure 26 shows the Fe^2+^ concentration in the solution and the XRD patterns of the minerals after 30 days of interaction between the mineral-modifying bacteria and the reservoir core powder. After 30 days of biological action, the Fe^2+^ concentration in the experimental group increased significantly, demonstrating a clear ability to reduce structural Fe^3+^ in montmorillonite, with an Fe^3+^ reduction efficiency of 36.57% to 37.49%. The XRD patterns indicate that after 30 days of action by the modifying bacteria, the characteristic XRD peaks of montmorillonite changed, with the disappearance of montmorillonite-specific peaks and the appearance of new mineral characteristic peaks around 2θ = 25°. This suggests the destruction of the montmorillonite structure and the occurrence of mineral phase transformation.

After 30 days of bacteria–mineral reaction, swelling rate analysis was conducted. The reaction system was transferred to a 50 mL centrifuge tube and allowed to stand for 72 h to observe the swelling volume of montmorillonite. Here, M-1 and M-2 are parallel experimental groups, while B-1 and B-2 are blank groups (B-1: no bacteria added; B-2: 50 mL of logarithmic phase bacterial solution centrifuged and inactivated to obtain dead bacterial cells). After 30 days of reaction under these conditions, the swelling reduction rate reached 24% to 47%.

**Figure 27:**
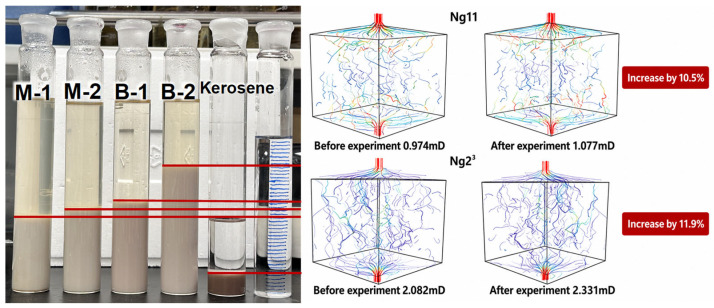
Shrinkage and swelling reduction effect of mineral-modified bacteria.

To quantitatively evaluate the impact of bacteria–mineral reactions on core permeability, permeability numerical simulations were conducted using Avizo 2020.1 based on pore network models reconstructed from CT scans. The simulated results are presented in Figure 27. The velocity streamline maps reveal significant differences in seepage field distribution among different samples, with pronounced heterogeneity in flow velocity along individual seepage paths: red streamlines correspond to the maximum velocity, while purple streamlines indicate the minimum. Overall, the boundary flow velocities at the inlet and outlet are markedly lower than those within the pore–fracture space; when the fluid passes through regions with irregular pore geometries or abrupt throat-radius constrictions, the velocity exhibits a distinct increase, consistent with the “vena contracta effect” in seepage mechanics. The calculated permeability values show that post-reaction core permeability increased from 0.974 mD and 2.082 mD to 1.077 mD and 2.331 mD, corresponding to enhancement magnitudes of 10.5% and 11.9%, respectively. These results confirm that microbial shrinkage and weak dissolution effects can stably achieve pore throat expansion and connectivity enhancement under varying porosity conditions, thereby effectively improving reservoir seepage capacity.

### 2.4. Field Synergistic Application Effects

To address the issue of severe velocity sensitivity, oil wells with daily fluid production below 6 t/d within two water injection well groups in the Jinjia oilfield were subjected to mixed displacement, acidizing, and sand control treatments to remove sand blockage around the wells (injecting 40 m^3^ of acid, 70 m^3^ of foam fluid for backflow flushing, 140 m^3^ of sand-carrying fluid, and 26 m^3^ of quartz sand). To tackle the problems of high crude oil viscosity and the tendency for asphaltene–clay interaction to cause formation blockage, a development test transitioning to bio-chemical desensitization and viscosity-reducing flooding was conducted. This was based on wellbore reconstruction and plug removal using a low-damage deep acidizing system. The injection method for the bio-chemical desensitization and viscosity-reducing agent solution was simulated using chemical reactions in CMG software. A “three-slug combined oscillatory injection method” was adopted, consisting of a primary test slug, a secondary main slug, and a tertiary protective slug. The injection concentration for the primary and tertiary slugs was 0.5–0.6%, with an injection volume of 0.05 PV each. The injection concentration for the secondary main slug was 0.9–1.0%, with an injection volume of 0.25 PV. The optimal timing for profile control was determined to be around 80% water cut, with a profile control agent concentration of 0.5% and a total injection volume of 30 t, achieving the best effect when the agent reached a position 0.3–0.4 of the distance between the injection and production wells.

Two injection wells were converted to bio-chemical desensitization and viscosity-reducing agent flooding, with a daily water injection rate of approximately 65 t/d, an injection concentration of 0.5–0.6%, and an injection pressure of approximately 5.6 MPa. The cumulative injection of bio-chemical desensitization and viscosity-reducing agent reached 388.3 t, with a cumulative injected solution volume of 7.35 × 10^4^ t. After conversion, due to the increased crude oil mobility, the oil production of the corresponding six production wells increased from 1.2 t/d to 3.3 t/d per well, representing a 2.8-fold increase in single-well production. Influenced by the decreased oil–water viscosity ratio, the water cut decreased significantly from 83.15% to 71.1%, a reduction of 12.05%. Compared with water flooding, the development performance improved markedly: the rate of water cut increase slowed down: the water cut increased at an average annual rate of 2% during the water flooding stage, whereas it decreased at an average annual rate of 1.78% after conversion to composite flooding. The incremental oil production increased substantially: during the water flooding stage, the cumulative water injection was 17.66 × 10^4^ t, yet the incremental oil production was only 3704 t; during the desensitization and viscosity-reducing agent flooding stage, the water injection was 7.35 × 10^4^ t, while the incremental oil production reached 9901 t. For the two pilot well groups, the economic benefits increased: the desensitization and viscosity-reducing agent stage incurred an additional investment of 3.97 million yuan, generated additional revenue of 23 million yuan, and achieved an input–output ratio of 5.79.

### 2.5. Application Value and Academic Contributions of the Research Achievements

In terms of application value, the bio-chemical composite flooding technology for desensitization and viscosity reduction developed in this study provides a replicable, low-cost and environmentally friendly development approach for ultra-sensitive heavy oil reservoirs represented by the Jinjia oilfield in Shengli oil province. This technology does not rely on steam thermal recovery, thus avoiding the technical bottlenecks of severe heat loss and low steam quality in thin reservoirs. Meanwhile, mineral-modified bacteria inhibit clay swelling from the source, solving the engineering problems of a short validity period and easy washout of chemical anti-swelling agents. Field tests demonstrate that this technology can convert sensitive heavy oil reservoirs with ineffective or low-efficiency water flooding into effective development units featuring both increased liquid production and enhanced oil output, with an input–output ratio of 1:5.79. The research outcomes can be directly extended to similar domestic ultra-sensitive heavy oil reservoirs, as well as heavy oil reservoirs with analogous geological conditions worldwide, possessing promising prospects for field application.

In terms of academic contributions, this study advances existing understandings in two aspects:

(1) For the first time, it systematically reveals the water–oil–solid coupled sensitization mechanism of ultra-sensitive heavy oil reservoirs. In particular, it verifies the positive feedback cycle of electrical adsorption between asphaltenes and clay minerals, particle size enlargement and viscosity rise, breaking through the limitation of traditional studies that treat water sensitivity and velocity sensitivity as independent influencing factors.

(2) A ternary synergistic composite oil recovery concept integrating biological desensitization, chemical viscosity reduction and gel profile control and flooding is proposed. Laboratory experiments and field tests validate its feasibility in ultra-sensitive reservoirs, expanding the application scope of microbial enhanced oil recovery technology. The research focus is extended from the conventional viscosity reduction and methanogenesis to mineral phase transformation modification and reservoir protection.

The above achievements are of positive significance for enriching the theoretical system of cold heavy oil production and promoting the development of enhanced oil recovery technologies for sensitive reservoirs.

## 3. Materials and Methods

### 3.1. Materials

The experimental oil utilized dehydrated crude oil from wellheads in the Es3 member of the Shengli Jinjia oilfield, as well as kerosene and naphthenic oil. The viscosity of the dehydrated degassed crude oil at 50 °C is 941 mPa·s. The experimental water employed was formation water, which is of the CaCl_2_ water type with a total salinity of 20,550 mg/L. The experimental chemicals used were self-developed systems. Water sensitivity tests utilized reservoir rock samples (500 mD). Particle migration tests and resistance coefficient tests employed artificial cores (30 cm, 500 mD/756 mD). Dual-tube experiments utilized sand-pack models (30 cm, 1472 mD and 328 mD). Swelling reduction experiments for bio-mineral modifying bacteria utilized montmorillonite mineral powder and core powder (200 mesh).

The experimental apparatus included a multifunctional core flooding apparatus, a swell meter, a zeta potential analyzer, a viscometer, a laser particle size analyzer, a scanning electron microscope (SEM), a micro-visualization device, and an X-ray diffractometer (XRD).

### 3.2. Experimental Methods

All experimental procedures and tests were conducted in accordance with relevant industry standards and technical specifications. Specifically, the sensitivity experiments were performed following the Chinese petroleum industry standard SY/T5358-2010 “Evaluation method for reservoir sensitivity flow experiments” [20]. To ensure the reliability and reproducibility of the results, all experiments, including sensitivity tests, core flooding experiments, zeta potential measurements, particle size distribution analyses, swelling coefficient tests, scanning electron microscopy (SEM) observations, and X-ray diffraction (XRD) analyses, were carried out in at least triplicate. All data are presented as mean ± standard deviation (SD). For key parameters, one-way analysis of variance (ANOVA) was used for significance testing, with a *p*-value < 0.05 considered statistically significant. All cores, fluids, and chemicals were sourced from the same batch, and experimental conditions (temperature, confining pressure, and flow rate) were strictly controlled. All raw data have been properly archived to ensure traceability.

Water sensitivity experiment steps: (1) Dry the core, weigh it, and measure the initial liquid permeability using standard brine. (2) Displace with 10–15 pore volumes (PV) of the intermediate test fluid. Maintain the confining pressure and temperature, allowing it to react with the rock minerals for over 12 h. Measure the permeability after the reaction. (3) Replace the fluid with distilled water and repeat step (2). (4) Record data such as pressure and flow rate during the experiment to calculate permeability and its rate of change.

Velocity sensitivity experiment steps: (1) Dry the core, weigh it, saturate it with standard brine, calculate porosity, and measure the initial liquid permeability. (2) Set different flow rates and displace with injection water. After the flow stabilizes, record parameters such as pressure and flow rate. (3) Calculate the permeability and its rate of change, and plot the curves.

Zeta potential experiment steps: (1) Prepare the test sample, disperse it in deionized water, and treat it with ultrasound. (2) Turn on the potentiometer and calibrate the electrophoretic mobility using a standard sample. (3) Select the Smoluchowski model, set the appropriate temperature, and set the number of measurements to three. Insert the sample cell, avoiding air bubbles. (4) After starting the measurement, record the electrophoretic mobility data, calculate the zeta potential using the Henry equation, and generate a potential distribution map.

Clay swelling coefficient experiment steps: (1) Sample preparation: weigh equal amounts of clay and place them into different centrifuge tubes. (2) Add kerosene, distilled water, and brines of different salinities to the respective centrifuge tubes and mix thoroughly. (3) Place the centrifuge tubes into the centrifuge for a set time. (4) Remove the tubes and record the clay volume in each tube. (5) Using the clay volume in the kerosene tube as the baseline, calculate the swelling multiples of the clay under different salinity conditions.

Laser particle size distribution experiment steps: (1) Take the produced fluid, add a dispersant, and stir evenly for later use. (2) Turn on the laser particle size analyzer and the circulation system, and set the test parameters. (3) Start the measurement program, inject the sample into the sample cell, maintain circulation and ultrasonic dispersion, and collect data.

Establishment of an experimental method for the water–oil–solid coupled sensitivity mechanism in ultra-sensitive reservoirs: (1) Multi-scale particle composite filling: based on the particle size analysis of reservoir rock samples, prepare clay of different mesh sizes (200–400 mesh) and quartz sand (40–100 mesh). Fill the prepared solid particles into a visual flat-panel model to ensure its pore structure closely resembles actual formation characteristics. (2) Multi-viscosity oil phase system preparation: using the oil phase as the base, prepare oil phases with different viscosities using green, non-toxic naphthenic oil and kerosene as viscosity stabilizers and regulators. (3) Multi-stage displacement dynamic simulation: simulate the migration characteristics of clay particles under displacement by different fluid media, based on actual reservoir development characteristics. (4) Threshold velocity determination: using different fluid media, conduct displacement experiments at various displacement rates to determine the flow rate at which inorganic particles begin to migrate as the threshold velocity. (5) Particle migration characteristic simulation: displace a set number of PV at the threshold velocity and observe the migration characteristics of inorganic particles. (6) Dynamic parameter monitoring: use high-precision pressure sensors and high-speed cameras to monitor pressure and images throughout the entire process. (7) Migration and plugging mechanism analysis: analyze the oil–solid interaction sensitivity mechanism by combining zeta potential and oil–solid adsorption property characteristics.

Scanning electron microscope (SEM) experiment steps: (1) Prepare the test sample (asphaltenes, clay minerals before and after swelling, etc.). (2) Place an appropriate amount of the sample on the sample stage and evacuate. (3) Locate the target area with low-magnification scanning, observe morphological changes before and after swelling at high magnification, and record backscattered electron or secondary electron signals. (4) Save comparative images before and after swelling, and measure changes in porosity or surface roughness.

The displacement experiment methods were based on SY/T6315-2017, “Method for measuring high-temperature relative permeability and oil displacement efficiency in heavy oil reservoirs.”

The microfluidic experimental procedures for water flooding and bio-chemical viscosity reduction flooding are as follows: (1) Chip preparation: select a microfluidic chip matching the pore throat characteristics of the target reservoir, clean and dry it, and measure and record the chip’s pore structure parameters. (2) Fluid preparation: prepare simulated formation water and the bio-chemical viscosity reduction composite system, and measure the viscosity, interfacial tension, and stability of each fluid. (3) Oil saturation: heat the heavy oil to a flowing state, inject it into the chip, and allow it to saturate at constant temperature; record the initial oil saturation. (4) Water flooding experiment: inject simulated formation water at a constant flow rate, acquire displacement front images in real time, record pressure changes and produced liquid volume, and terminate when the water cut reaches 98% or the pressure stabilizes. (5) Bio-chemical flooding experiment: switch to injecting the composite system, control the injection rate and slug size, and dynamically monitor the displacement morphology, pressure response, and changes in residual oil distribution. (6) Post-flush water flooding: conduct water flooding again after composite system injection to evaluate the enhanced oil recovery factor, and compare and analyze the microscopic oil displacement efficiency differences between water flooding and bio-chemical flooding. (7) Image analysis: after the experiment, use image processing software to quantitatively analyze the oil saturation, displacement front uniformity, and residual oil mobilization degree at each stage.

Test procedures for crude oil viscosity at different temperatures: (1) Sample preparation: heat crude oil to a flowing state, stir thoroughly, and remove water and impurities. (2) Instrument setup: power on and preheat for 30 min; select measuring system based on viscosity; complete calibration. (3) Loading and thermal equilibration: load sample and hold at target temperature for 10–20 min with deviation within ±0.1 °C. (4) Pre-shear: pre-shear at high shear rate (60–120 s) to eliminate thixotropic effects, then rest for 5–10 min for structure recovery. (5) Viscosity measurement: perform shear rate sweep, record shear stress, and calculate apparent viscosity. (6) Repeat the steps of equilibration, pre-shear, and measurement at each set temperature; run 2–3 replicates per temperature and average. (7) Data processing: plot viscosity–temperature curves, fit the Arrhenius equation, and analyze viscosity–temperature characteristics and Newtonian/non-Newtonian behavior.

Synthesis procedure of the bio-chemical desensitization and viscosity-reduction agent: (1) Into a 500 mL four-necked round-bottom flask equipped with a mechanical stirrer, nitrogen inlet tube, constant-pressure dropping funnel, and reflux condenser, 250 mL of anhydrous ethanol was added as solvent. Acrylamide (AM, 5.32 g), rhamnosyl acrylate (Rha-AC, 6.48 g), styrene (St, 1.04 g), and 2-(methacryloyloxy) ethyl dimethyl-(3-sulfopropyl) ammonium betaine (DMAPS, 1.41 g) were added sequentially, and the mixture was stirred at room temperature for 30 min until complete dissolution. (2) Nitrogen was bubbled through the solution for 30 min to remove dissolved oxygen, and the temperature was raised to 65 °C. Azobisisobutyronitrile (AIBN, 0.15 g, 0.8 wt% of total monomer mass, dissolved in 10 mL ethanol) was added, and the reaction was carried out at 65 °C under nitrogen protection for 10 h. (3) After completion of the reaction, the polymer solution was slowly poured into acetone (1500 mL) to precipitate the polymer. The precipitate was filtered and washed with acetone three times (100 mL each), then vacuum-dried at 50 °C for 48 h to afford a white to pale yellow flocculent solid.

Synthesis procedure of the self-growing gel dispersion profile control agent: (1) In situ preparation of sodium acrylate: acrylic acid (12.0 g) was dissolved in 50 mL water, and NaOH solution (4.8 g dissolved in 20 mL water) was slowly added dropwise under an ice-water bath, maintaining the temperature below 25 °C and neutralizing to pH ≈ 7.0. (2) Into a 1 L four-necked round-bottom flask, the following were added sequentially: acrylamide (AM, 85.0 g), sodium acrylate solution (NaAA, 12.0 g), catechol-structured monomer (DOPA-AC, 3.0 g), chain transfer agent (PAM-X, 0.35 g), and anhydrous ethanol/water mixed solvent (500 mL). The mixture was stirred at room temperature for 30 min until complete dissolution, and nitrogen was bubbled for 30 min to remove dissolved oxygen. (3) AIBN (0.012 g, dissolved in 10 mL ethanol) was added, the temperature was raised to 65 °C, and the reaction was carried out under nitrogen protection for 12–16 h (conversion > 90%). (4) The reaction solution was slowly poured into acetone (5 L) to precipitate the polymer, filtered, and washed with acetone three times (200 mL each), then vacuum-dried at 50 °C for 48 h to afford a white to light brown flocculent solid CFPAM. (5) CFPAM (0.35 g) was added to deionized water (98.6 g) and stirred at room temperature until complete dissolution to obtain a homogeneous solution. Water-soluble phenol-formaldehyde resin (1.0 g) was added and stirred at room temperature for 15 min until fully miscible. The mixed solution was transferred to a sealed container and aged in an oven at 80 °C for 60 h to obtain a bulk hydrogel, appearing as a translucent to light brown elastic solid.

Oil displacement experiment steps: (1) Dry the rock sample, weigh it, saturate it with water, and measure initial permeability and porosity. (2) Saturate with oil and age at formation temperature and pressure. (3) Perform water flooding at the set injection rate until a high water cut stage is reached. (4) Switch to chemical flooding and inject a set amount of the profile control and displacement system. (5) After injecting the chemicals, switch to subsequent water flooding. (6) Record data such as pressure and flow rate during the experiment.

Experimental procedure for bacterial isolation: The mineral-modifying bacteria were directionally isolated using an in situ enrichment-gradient selection method to selectively screen for thermophilic iron-reducing and decomposing microbial consortia, and a bacterium–mineral dynamic reaction system was established with disodium nucleotide as the sole phosphorus source. First, the concentrated iron-reducing bacterial isolation medium was prepared, adjusted to pH 6.5 ± 0.2, dispensed into 115 mL anaerobic bottles, and autoclaved at 121 °C for 20 min. In the experimental group, 80 mL of oilfield produced water containing the indigenous microbial community was injected; the control group received an equal volume of sterile ultrapure water instead. Both groups were incubated under anaerobic conditions at 63 ± 2 °C with shaking at 50 rpm. The specific workflow comprised: (1) preparation of concentrated medium; (2) strict anaerobic inoculation; (3) dynamic cultivation and monitoring; (4) enrichment, screening, and preservation of dominant consortia; and (5) establishment of the bacterium–mineral dynamic reaction system, followed by periodic sampling and testing, and subsequent characterization of mineral composition and elemental content.

Mineral composition detection was based on SY/T 5163-2018, “Analysis method for clay minerals and common non-clay minerals in sedimentary rocks by x-ray diffraction.” Experimental steps: (1) Extract clay minerals from sedimentary rocks, grind them to a particle size of less than 2 μm, and prepare oriented thin film samples. (2) After preheating, set the scanning range and accelerating voltage. (3) Scan the sample to obtain a diffraction pattern and qualitatively analyze the mineral types based on characteristic peaks. (4) Calculate the relative mineral content using the K-value method or internal standard method.

Avizo numerical simulation procedure: (1) Model preprocessing: based on the 3D voxel-reconstructed model, pore-matrix phase segmentation and denoising were performed using the “Volume edit” and “Label analysis” modules to ensure the geometric continuity and topological connectivity of the pore network. (2) Boundary condition setup: fluid flow was simulated as one-dimensional steady-state laminar flow along the Z-axis, with no-slip wall conditions applied; the side walls were sealed and isolated such that there was no leakage of internal fluid or intrusion of external fluid. A pressure gradient was used to drive the seepage, with the inlet pressure set to 1.1 MPa and the outlet pressure set to 0.1 MPa. (3) Solver and convergence control: the velocity field and pressure drop distribution within the pores were calculated based on a steady-state laminar flow model. The convergence coefficient was set to 0.0001, with a minimum of 1000 iterations and a maximum of 10^6^ iterations. Finally, the volume-averaged permeability was calculated according to Darcy’s law.

## 4. Conclusions

The water–oil–solid coupled sensitivity mechanism in sensitive reservoirs was clarified. Water–solid swelling: Montmorillonite absorbs water, leading to structural instability, dispersion, and migration. Oil–solid agglomeration: Particle–asphaltene adsorption and asphaltene aggregation increase particle size, causing pore throat blockage. Oil–solid entrainment: Mineral particles are entrained and migrated by high-viscosity fluids, resulting in plugging. Water–oil–solid coupling: water and oil carry migrated particles, forming localized high-permeability channels.

A novel composite system, including a bio-chemical desensitization and viscosity-reducing agent and a self-growing gel dispersion profile control agent, was developed by integrating biological and chemical methods. Functional bacteria for modifying sensitive minerals promote the transformation of montmorillonite to illite by reducing structural iron, achieving fundamental desensitization. Through functional chemical actions such as emulsification viscosity reduction, oil stripping, and emulsion droplet plugging, the system synergistically addresses the challenges of sensitivity plugging and channeling. This enables synergistic enhancement in the novel viscosity-reducing composite flooding system via bio-chemical desensitization and viscosity reduction, clay stabilization during injection and production operations, and profile control using the self-growing gel dispersion for injection-production regulation. The recovery rate of the composite drive can reach 56.5%, which is 26.3% higher than that of the post-water drive demulsification drive.

After converting two pilot well groups in Jinjia oilfield from water flooding to bio-chemical desensitization and viscosity-reducing composite flooding, the oil production capacity per well increased by 2.8-fold, the water cut decreased by 12%, and both the development performance and economic benefits improved significantly, achieving a transformation from “increasing water without increasing oil” to “increasing both liquid and oil production.”

Due to the limited availability of experimental samples, this study has constraints in its experimental design and has not yet systematically evaluated measurement uncertainty and experimental repeatability. Future work will include replicate measurements (at least triplicates per condition) to quantify errors introduced by temperature control, instrument precision, and operational factors, thereby establishing a systematic error assessment framework to enhance data reliability.

## Figures and Tables

**Table 1:** Data table of particle initiation velocities under different fluid viscosities.

Viscosity (mPa·s) 25 °C	Threshold Velocity of Inorganic Particles (mL/min)					
	Sodium-Based Montmorillonite	Calcium-Based Montmorillonite	Illite	Potassium Feldspar	Albite	Quartz
0.89	10	50	20	100	20	20
110	2	5	5	5	5	5
560	1	2	3	2	3	4

**Table 2:** Six desensitizing viscosity-reducing oil displacement agents with different degrees of polymerization.

Number	Desensitization Rate %	Adsorption Rate %	IFT mN/m	Viscosity Reduction Rate %	Viscosity Reduction Rate After Adsorption %	Oil Washing Efficiency %	Dehydration Rate by Natural Sedimentation %	pH	Range of Oil–Water Ratio %					Viscosity Reduction Concentration Window %			
									8:2	7:3	5:5	3:7	2:8	0.2	0.3	0.4	0.5
1	40.6	1.54	4.1 × 10^−2^	93.2	92.1	66.9	81.5	6.5	90.5	93.2	96.8	97	98.2	92.3	93.2	95.8	96
2	42.4	1.61	3.6 × 10^−1^	93.4	92.6	70.8	85.1	6.5	90.6	93.4	96.5	97.1	98.5	93.3	93.4	96.2	97
3	36.1	1.57	8.6 × 10^−1^	93.4	92.9	66.7	85.8	6	90.9	93.4	96.9	97.5	98.7	93.1	93.4	96.1	97.1
4	49.7	2.31	1.7 × 10^−1^	90.2	86.3	71.8	81	8	89.8	90.2	93.8	96	99	90	90.2	89.8	91
5	56.0	1.19	1.0 × 10^−3^	99.1	98.9	68	94.4	7	97.7	99.1	98.8	99	99.1	99.3	99.1	99.3	99.4
6	39.5	1.17	6.4 × 10^−3^	99.5	98.2	72.8	95.5	6	97.5	99.5	98.9	99.1	99.2	98.5	99.5	99.5	99.5

## Data Availability

The original contributions presented in this study are included in the article. Further inquiries can be directed to the corresponding author.
